# Validation of the angular measurements of a new inertial-measurement-unit based rehabilitation system: comparison with state-of-the-art gait analysis

**DOI:** 10.1186/1743-0003-11-136

**Published:** 2014-09-11

**Authors:** Alberto Leardini, Giada Lullini, Sandro Giannini, Lisa Berti, Maurizio Ortolani, Paolo Caravaggi

**Affiliations:** Movement Analysis Laboratory, Istituto Ortopedico Rizzoli, Bologna, Italy

**Keywords:** Inertial measurement unit, Gait analysis, Rehabilitation, Knee, Hip, Thorax, Joint flexion, Audio-visual bio-feedback, Video-games

## Abstract

**Background:**

Several rehabilitation systems based on inertial measurement units (IMU) are entering the market for the control of exercises and to measure performance progression, particularly for recovery after lower limb orthopaedic treatments. IMU are easy to wear also by the patient alone, but the extent to which IMU’s malpositioning in routine use can affect the accuracy of the measurements is not known. A new such system (Riablo™, CoRehab, Trento, Italy), using audio-visual biofeedback based on videogames, was assessed against state-of-the-art gait analysis as the gold standard.

**Methods:**

The sensitivity of the system to errors in the IMU’s position and orientation was measured in 5 healthy subjects performing two hip joint motion exercises. Root mean square deviation was used to assess differences in the system’s kinematic output between the erroneous and correct IMU position and orientation.

In order to estimate the system’s accuracy, thorax and knee joint motion of 17 healthy subjects were tracked during the execution of standard rehabilitation tasks and compared with the corresponding measurements obtained with an established gait protocol using stereophotogrammetry.

**Results:**

A maximum mean error of 3.1 ± 1.8 deg and 1.9 ± 0.8 deg from the angle trajectory with correct IMU position was recorded respectively in the medio-lateral malposition and frontal-plane misalignment tests. Across the standard rehabilitation tasks, the mean distance between the IMU and gait analysis systems was on average smaller than 5°.

**Conclusions:**

These findings showed that the tested IMU based system has the necessary accuracy to be safely utilized in rehabilitation programs after orthopaedic treatments of the lower limb.

**Electronic supplementary material:**

The online version of this article (doi:10.1186/1743-0003-11-136) contains supplementary material, which is available to authorized users.

## Background

Biofeedback has been used extensively in physical medicine and rehabilitation of human joints to facilitate recovery to normal function after injury and treatments [1]. Audio and visual feedbacks are intended to encourage patients to perform rehabilitation exercises with more attention, more accurately, and more frequently by adding entertainment to the execution of physical exercises. The signals on the position and orientation of the body segments involved in the movement exercise should provide users with valuable feedback on the quality of their performance. This can be displayed in the basic form of numbers (direct inclinations or joint angles, general scores, etc.), geometrical entities or simple bar plots [2], up to complete immersive virtual environments typical of video-games [3–7].

Since manual and physical-exercise based physiotherapy provided in standard rehabilitation centres entails great expenses and resources, the use of self-administered training systems, which can be used at the patient’s home, is being investigated [8, 9]. These modern rehabilitation systems are highly portable, easy to use, and with a friendly graphical restitution, which is expected to facilitate the effective execution of standard and novel rehabilitation programs. Most of such systems are based on relatively low-cost inertial measurement units (IMU), which have been shown to be robust, small, and light to be worn on relevant body segments [1, 10, 11]. Typical target patients are those recovering from lower limb injury or joint reconstructions, these being usually adults keen to perform physical exercises at home [12, 13]. While, on the one hand, a home-based rehabilitation program offers several advantages in terms of costs involved and convenience for the patient [14], on the other it is more subjected to human error that may hinder the correct application of the protocol and thus decrease its value.

Recently, a new such rehabilitation system has been developed and initially configured for the functional recovery of the lower limb joints. However, incorrect positioning of the IMU on the body segments in unsupervised utilization can hinder the system’s performance, therefore its sensitivity in tracking joint rotations to known IMU’s malposition and in standard end-user settings must be assessed. The aim of this study was to assess the system’s reliability and accuracy during standard physical exercises using stereophotogrammetry as gold-standard.

## Methods

### The IMU based rehabilitation system

The Riablo™ (CoRehab, Trento, Italy) is an adaptive system, comprised of several IMU connected wirelessly to a computer, developed to enhance standard rehabilitation programs by guiding the user in performing prescribed physical exercises through a video interface. The IMU used weighs 20 grams, is based on the wireless Bluetooth™ communication protocol, and works at a sampling frequency of 50 Hz. Nine degrees of freedom are provided by the following sensors: a 3D accelerometer at ±2g full-scale, a 3D gyroscope at ±2000 dps full-scale, and a 3D magnetometer at ±1000 μT full-scale. The IMU sensors are calibrated at the factory before delivery.

**Figure 1:**
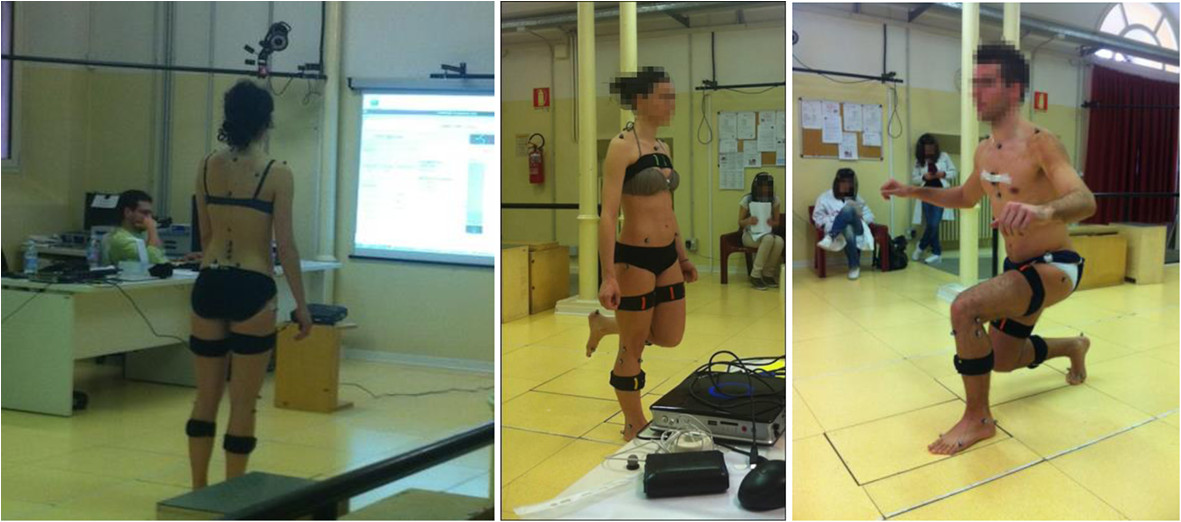
**Pictures of the data collection.** Two volunteers instrumented with IMU and markers for gait analysis during data collection in the gait laboratory; up-right posture (left), knee flexion against gravity (centre), lunge (right) are shown.

Simple videogames provide audio and visual feedbacks to guide the subject in performing the rehabilitation exercises while wearing light IMU in a number of body segments. The units are self-worn on the frontal aspect of body segments via elastics bands. For the present study, shanks, thighs and thorax were instrumented to track knee and hip joint motion and the thorax inclination during the exercises (Figure 1).

The rotation angles are computed through a proprietary algorithm based on the Kalman filter theory [15]. Accordingly, a different weight is given to the position and orientation signals from the accelerometer (k_a_), the gyroscope (k_g_) and the magnetometer (k_m_), so that the sum k_a_ + k_g_ + k_m_ is equal to 1. The weighted collected signals are fused to provide a measure of the overall spatial orientation (pitch, roll, yaw) for each IMU.

A software calibration algorithm removes any offset associated to initial misalignments, typically due to IMU malpositioning and/or to the body segment peculiar shape. On screen instructions and recommendations help the user to limit the former as much as possible. Simple images show the user how to wear the elastic bands appropriately on the body segments, and to place the IMUs in the correct pouches according to color- and numerical- codes. A static calibration, which entails the user to maintain a double-leg up-right posture for a few seconds, is required to measure the neutral joint position between IMUs, according to standard angle calculation. This is assumed to be the initial offset to be used then in each dynamic exercise.

### IMU sensitivity

The effects of different combinations of k weights on the kinematic-output were evaluated in a knee flexion-extension exercise performed by one subject. For the optimal triplet of k weights, the system sensitivity to IMU malposition was assessed via two tests. One test aimed at assessing the effects on the calculated joint rotations for three frontal-plane orientations of the IMU (0° correct; -15° and +15°) within the elastic band in a hip abduction/adduction exercise (target abduction angle = 35°). Another test aimed at assessing the effects on the calculated joint rotations due to three medio-lateral positions of the IMU (correct, -7cm and +7cm) in a hip flexion/extension exercise (target flexion angle = 90°). Both tests were performed by five healthy male subjects (25-35 years; 68-80 kg; 165-190 cm) each wearing three sets of IMU on the leg and thorax, for the three different configurations to be tested simultaneously. Root mean square deviation (RMSD) of the rotation trajectories over exercise duration in relation to those in the optimal IMU position/orientation was used to estimate the system’s sensitivity to IMU malpositioning.

### System accuracy

**Figure 2:**
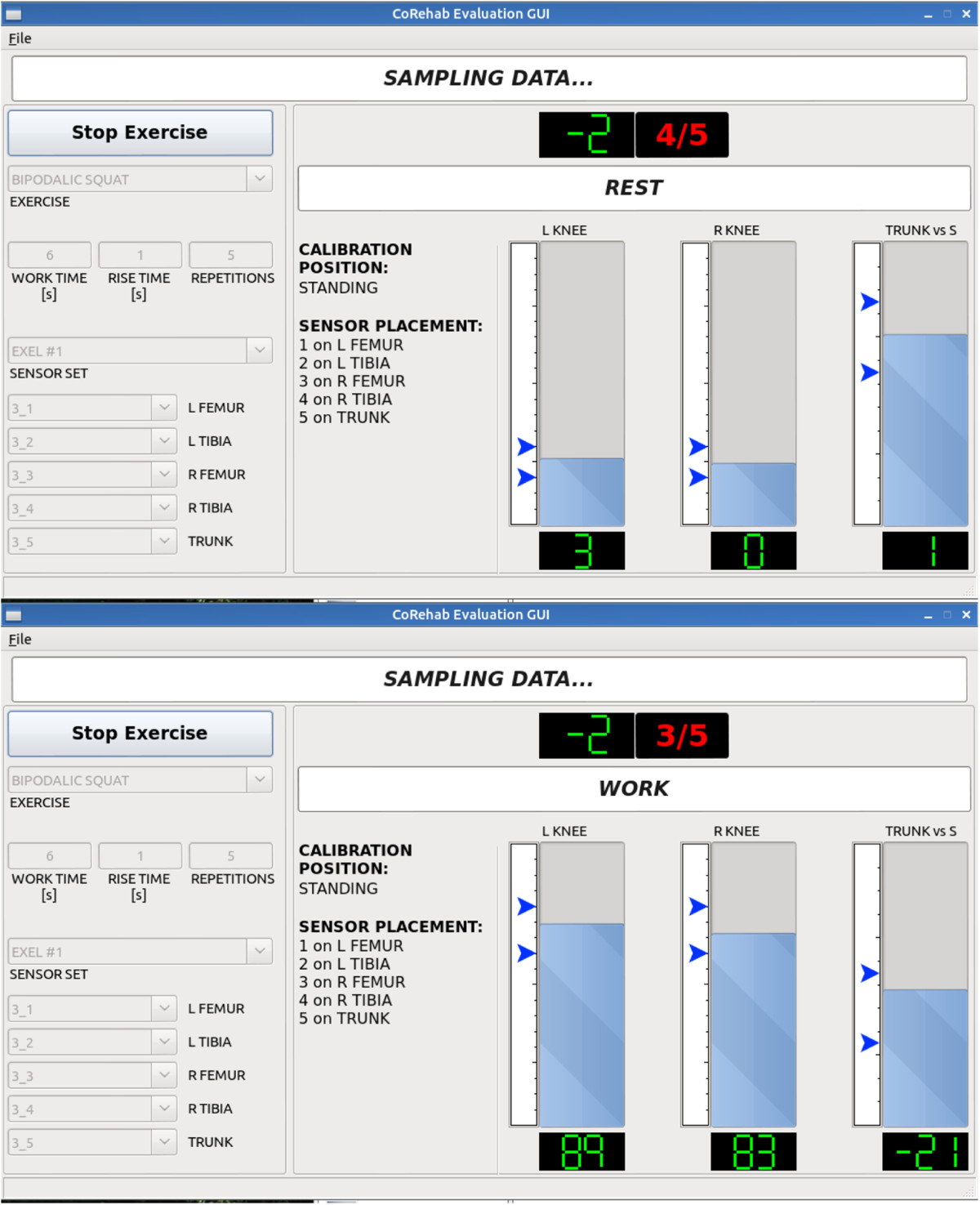
**Screenshots of the visual feedback.** Exemplary screenshots of the special interface used as visual feedback during the rest (top) and work (bottom) phases of the squat exercise. Users can monitor in real-time the knee (L KNEE and R KNEE) and thorax (TRUNK vs S, lunge and squat exercises only) flexion angles via bar plots and numerical values, and the desired target range by the position of the blue arrows. They can also have access (top of the screen) to the countdown (in green) and the repetition (red).

The system accuracy in standard simulated rehabilitation activities was also tested in 17 healthy young adults (10 men, 7 women; age 26.3 ± 3.8 years; height 176.1 ± 8.4 cm; weight 69.6 ± 11.8kg; BMI 22.3 ± 2.3), who volunteered for the study (Figure 1). The subjects were first instructed on how to wear the five elastic bands and relevant IMUs on the two thighs, shanks and on the thorax. These instructions were meant to simulate the initial training with the therapist as to make the user autonomous in operating the system. The following four exercises, typical of many rehabilitation programs for the knee joint, were performed on both left and right leg: lunge; knee flexion against gravity in single leg up-right posture; knee extension against gravity from the chair, and squatting. Five repetitions were recorded for each subject performing each exercise. The entire procedure, from IMU mounting to complete data collection, was repeated two times per each of the 17 subjects. In addition, up-right static posture was collected. The overall quality of the exercise was assessed through the analysis of knee joint rotations and thorax orientation, as recorded by the five IMU. In particular, in each exercise the targeted range of knee joint flexion was tracked by the thigh and shank IMU. For the lunge and squat only, as recommended by the specialists, the sagittal-plane inclination of the thorax was also tracked by the corresponding IMU. The IMU calculated angles, along with the targeted range, were displayed to the subject in real-time via a simple visual interface (Figure 2). The sequence of exercises, the target range of motion, and the rest time periods were also configured in the system and displayed to the user.

Simultaneously, three-dimensional rotations of the knee and thorax were measured via standard gait analysis (GA) system. Before starting the data collection, spherical 15-mm reflective markers were located on the lower limbs, pelvis and thorax according to validated protocols [16, 17], and tracked at 100 Hz during the exercise via an 8-TV-camera stereophotogrammetric system (Vicon motion systems, UK). These markers established anatomical-based reference frames, from which knee flexion/extension and thorax inclination in the sagittal plane were determined according to international recommendations [18]. Motion of the thigh with respect to the shank, and of the thorax with respect to the laboratory in the sagittal plane only (i.e. flexion), were used as gold-standard for the corresponding IMU measurements. Synchronisation between IMU and GA measurements was achieved a-posteriori from visual inspection of the rotation patterns.

## Results

### IMU sensitivity

**Figure 3:**
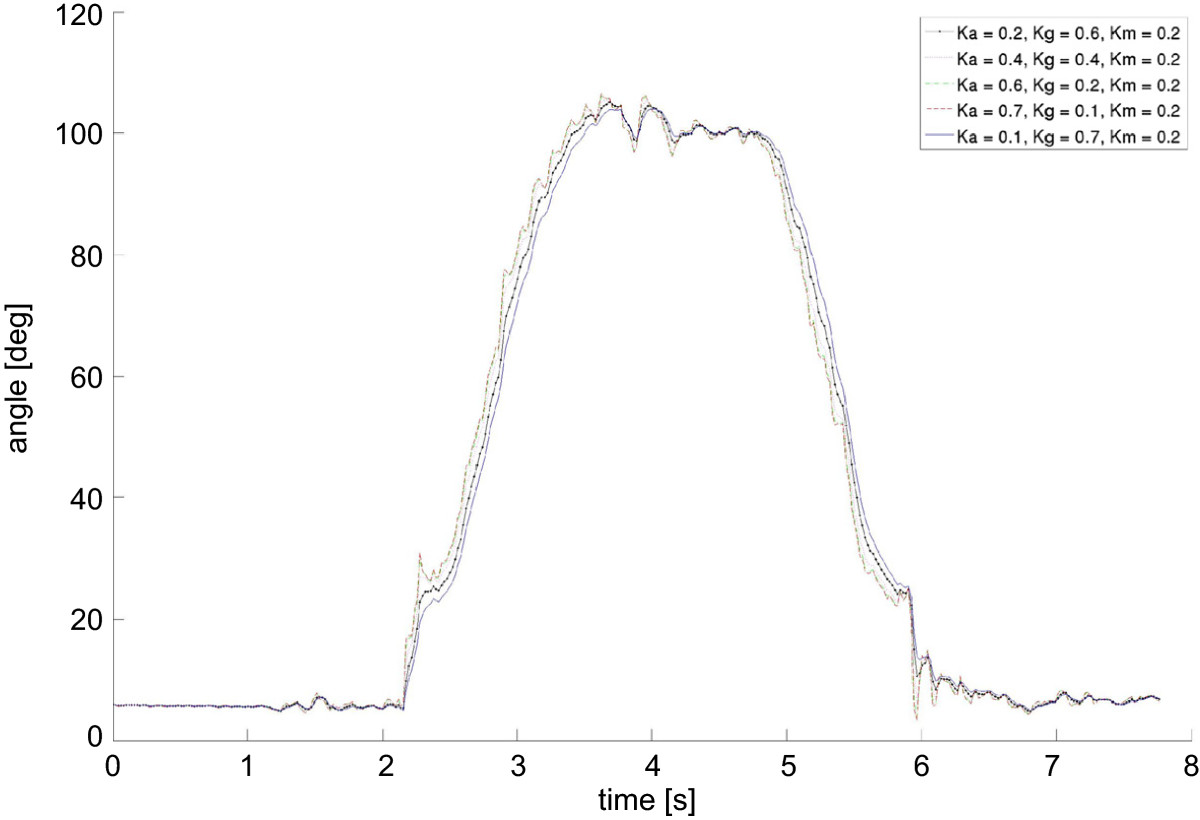
**Kalman filter weights.** Superimposition of flexion angle trajectories in a knee flexion against gravity exercise for different combinations of Kalman filter weights. Where k_a_, k_m_ and k_g_ are the filter’s weights of the accelerometer, magnetometer and gyroscope respectively.

**Figure 4:**
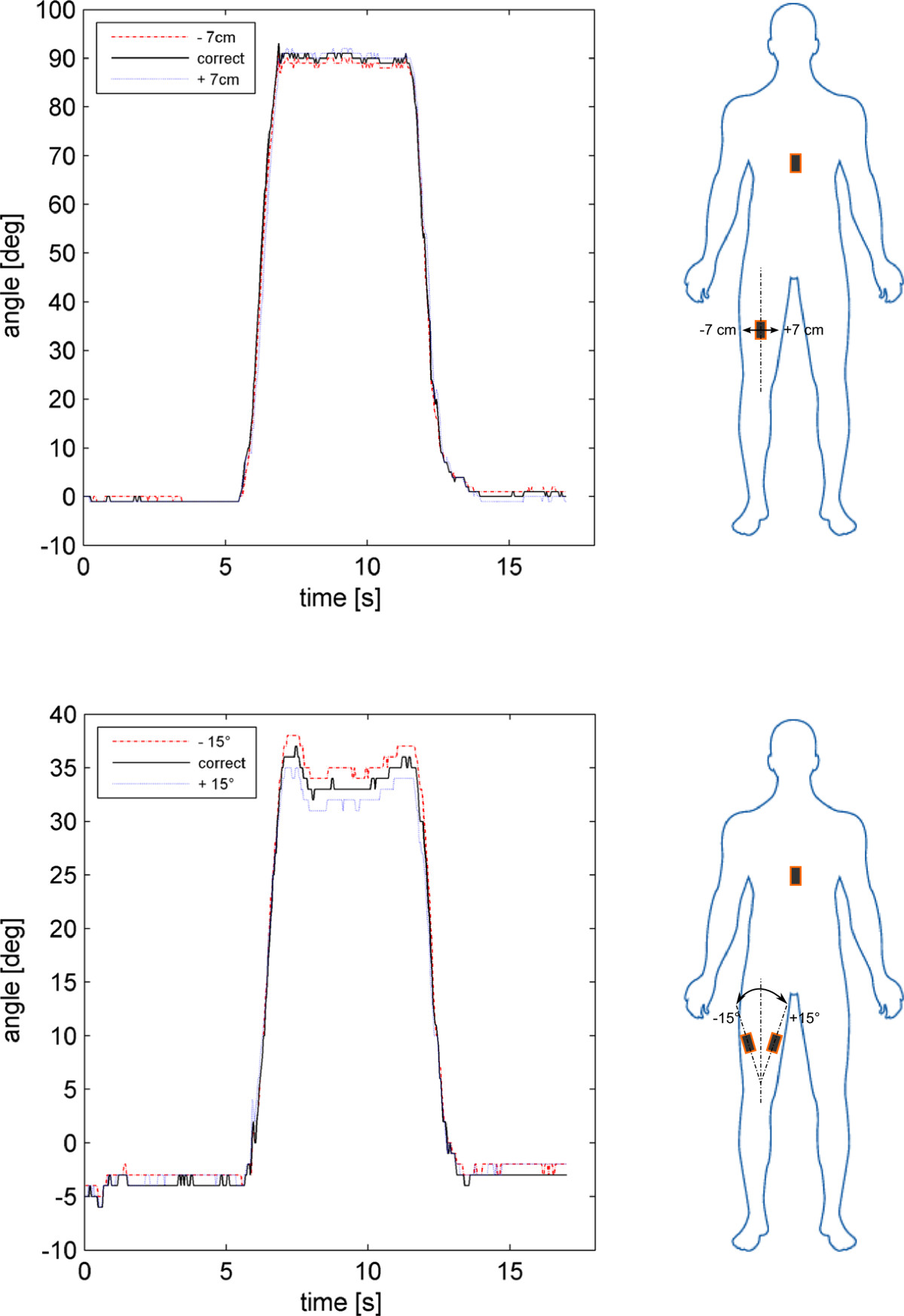
**System sensitivity to IMU malposition.** Top, superimposition of the kinematic trajectories [deg] from three pairs of IMU for a hip flexion/extension exercise in one subject. The three pairs of IMU were worn simultaneously on the subject’s leg and thorax, each in one of the three positions of -7 cm, 0 cm, and +7 cm with respect to the correct medio-lateral position. Bottom, superimposition of the kinematic trajectories [deg] from three pairs of IMU for a hip abduction exercise in one subject. The three pairs of IMU were worn simultaneously on the subject’s leg and thorax, each in one of the three orientations of -15°, 0° and 15° with respect to the correct frontal-plane orientation.

The optimal triplet of k weights ensuring a good compromise between output-signal smoothness and high responsiveness was: k_a_ = 0.2 ; k_g_ = 0.6, and k_m_ = 0.2 (Figure 3). With this triplet of weights for the Kalman filter, the sensitivity of the system to IMU malpositioning was calculated. In the medio-lateral IMU configurations test (Figure 4, top), the RMSD of the output rotation trajectories in the -7 and +7 cm configuration in relation to the optimal IMU position was, respectively, 2.1 ± 1.5 deg and 3.1 ± 1.8 deg across the 5 subjects. In the frontal-plane test (Figure 4, bottom), the RMSD of the output rotation trajectories in the -15 and +15 deg configuration in relation to the optimal IMU orientation was, respectively, 1.3 ± 0.6 deg and 1.9 ± 0.8 deg across the 5 subjects.

### System accuracy

**Figure 5:**
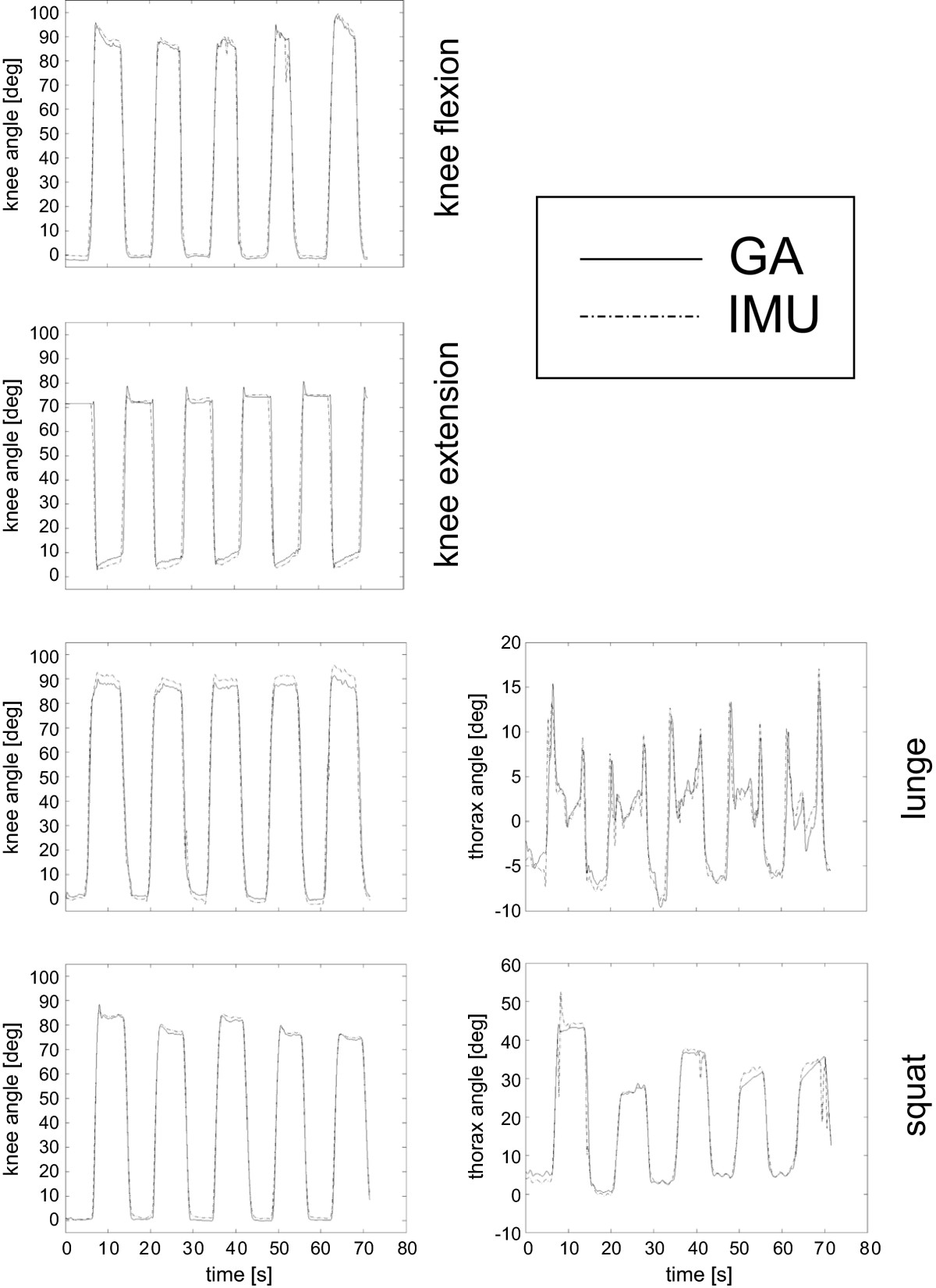
**Superimposition of GA and IMU measurements of knee and thorax flexion patterns.** Superimposition of IMU and corresponding GA flexion angles of the knee (left column) and thorax (right column) over each exercise (rows) in a typical subject.

The GA and IMU calculated knee flexion and thorax inclination angles were superimposed for each exercise (see Figure 5). In the squat exercise (bottom, Figure 5), five repetitions of about 80° knee flexion were performed in about 70 seconds, along with thorax motion in the sagittal plane between 0° and 45°. In the knee, a small bias can be observed in knee flexion and squat exercise, and a little larger range of flexion is calculated by the IMU system in knee extension and lunge exercises. With the exclusion of a few spikes from the IMU, thorax motion compared well across the exercises.

The mean distance between the joint rotation angles recorded by GA and IMU systems was evaluated for each subject and each exercise and averaged over the subjects (Tables 1 and 2). Mean rotation difference was smaller than 5 and 3 degrees respectively for knee flexion and for thorax inclination. The best (minimum distance) and the worst (maximum distance) measure for each exercise is also reported.

**Table 1:** **Knee error**

Exercise:	Knee flexion			
	*Target ROM*	*Mean distance*	*MIN mean dist.*	*MAX mean dist.*
Knee flexion	0 - 95	3.9 ± 0.7	2.2	6.1
Knee extension	0 - 90	3.8 ± 0.8	1.8	5.9
Lunge	0 - 100	4.5 ± 1.3	2.0	7.9
Squat	0 - 100	5.0 ± 1.2	2.4	8.3

Differences between IMU and GA for knee flexion angles [deg] over the 17 subjects analyzed.

**Table 2:** **Thorax error**

Exercise:	Thorax inclination			
	*Target ROM*	*Mean distance*	*MIN mean dist.*	*MAX mean dist.*
Lunge	0 - 25	1.6 ± 0.6	0.6	3.8
Squat	0 - 45	2.7 ± 2.1	0.6	7.5

Differences between IMU and GA for thorax inclination [deg] over the 17 subjects analyzed.

## Discussion

The Riablo system was developed to enhance physical rehabilitation by motivating the user in the execution of prescribed exercises either under the supervision of the physiotherapist or independently at home. Simple videogames provide audio and visual feedback according to the orientation and movement of light IMU worn on relevant body segments. Type and difficulty of the videogames were designed by specialists to address different rehabilitation needs.

New measurement units for human segment and joint motion should be validated before being introduced into the clinical setting. Recently, this has been performed for a novel motion tracking systems originally designed for video-games [19]. Several original IMU-based techniques to track lower limb joints motion have been proposed [10, 11, 20, 21], but only a few have been validated using stereophotogrammetry as the gold-standard [19, 22–28], as performed in the present study. As expected, the knee flexion angle was found to be the best to be estimated by the IMU among the three rotations [25].

In the present study, the sensitivity of the system to errors in IMU positioning in measuring joint angle trajectories appeared to be acceptable in the scenario of typical lower limb rehabilitation programs. While only a few erroneous IMU configurations were tested in this study, and no combinations of mal-orientation and mal-position were evaluated, the extent of erroneous malpositioning in routine usage is limited by the conforming shape of the pouch carrying the IMU in the elastic band. Moreover, absolute IMU deviations from the correct vertical alignment larger than 15° result in the calibration process to fail and a warning message being displayed to the user to correct the IMU position. As for the system’s accuracy, the knee joint angles calculated by the IMUs compared very well with those obtained from gait analysis based on stereophotogrammetry, though these IMUs were self-worn by the subjects as in the actual rehabilitation settings. Similarly, the thorax flexion was found to be well estimated by the corresponding IMU, as already reported in the relevant literature [22, 29].

It should be highlighted that the high quality/resolution of the videogames, normally used to guide the users to perform the exercises for this system, would have required a relatively low data collection sampling rate. Therefore, for the present validation study, special audio and visual feedbacks (Figure 2) were used to allow the IMU system’s sampling frequency to better match the 100 Hz of the stereophotogrammetric system. Deviations from the targeted degrees of knee flexion may be considered acceptable for these rehabilitation exercises to be safe to the patient.

While no major differences are expected to be found in patients after standard orthopaedic treatments of the lower limb joints, the present study is limited by the population of young and healthy subjects analysed. The influence of severe knee deformities on the calculated joint flexion/extension trajectories and the accuracy in tracking other segments and joints should be investigated separately in future studies. Finally, the gait analysis technique adopted might have its own limitations and different definitions, but it is among the most complete and validated, designed according to international standards in biomechanics.

## Conclusions

The present work investigated the sensitivity and accuracy of a modern rehabilitation system, in particular the angular measurements at the knee and thorax were compared with the corresponding measurements from state-of-the-art gait analysis. The results showed that the IMU based system has small errors in measuring joint rotations even in the present self-worn condition. The system appears therefore suitable to be used in routine rehabilitation of the lower limb joints, following orthopaedic treatment or during recovery from injury, also in a self-administered home setting.

## Authors’ original submitted files for images

Below are the links to the authors’ original submitted files for images. Authors’ original file for figure 1 Authors’ original file for figure 2 Authors’ original file for figure 3 Authors’ original file for figure 4 Authors’ original file for figure 5
