# Supplementary figures and images for: Validation of the angular measurements of a new inertial-measurement-unit based rehabilitation system: comparison with state-of-the-art gait analysis
Source: J Neuroeng Rehabil. 2014 Sep 11;11:136. doi: 10.1186/1743-0003-11-136 (PMC4169865; doi:10.1186/1743-0003-11-136)

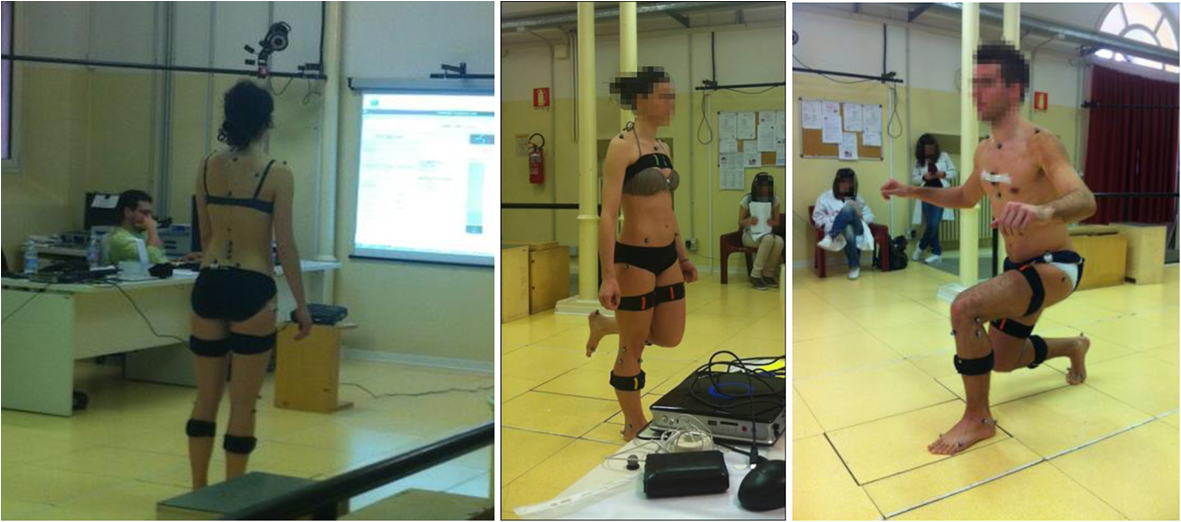

Supplement: Supplementary file 1 — Authors’ original file for figure 1 [file 12984_2014_656_MOESM1_ESM.tif]

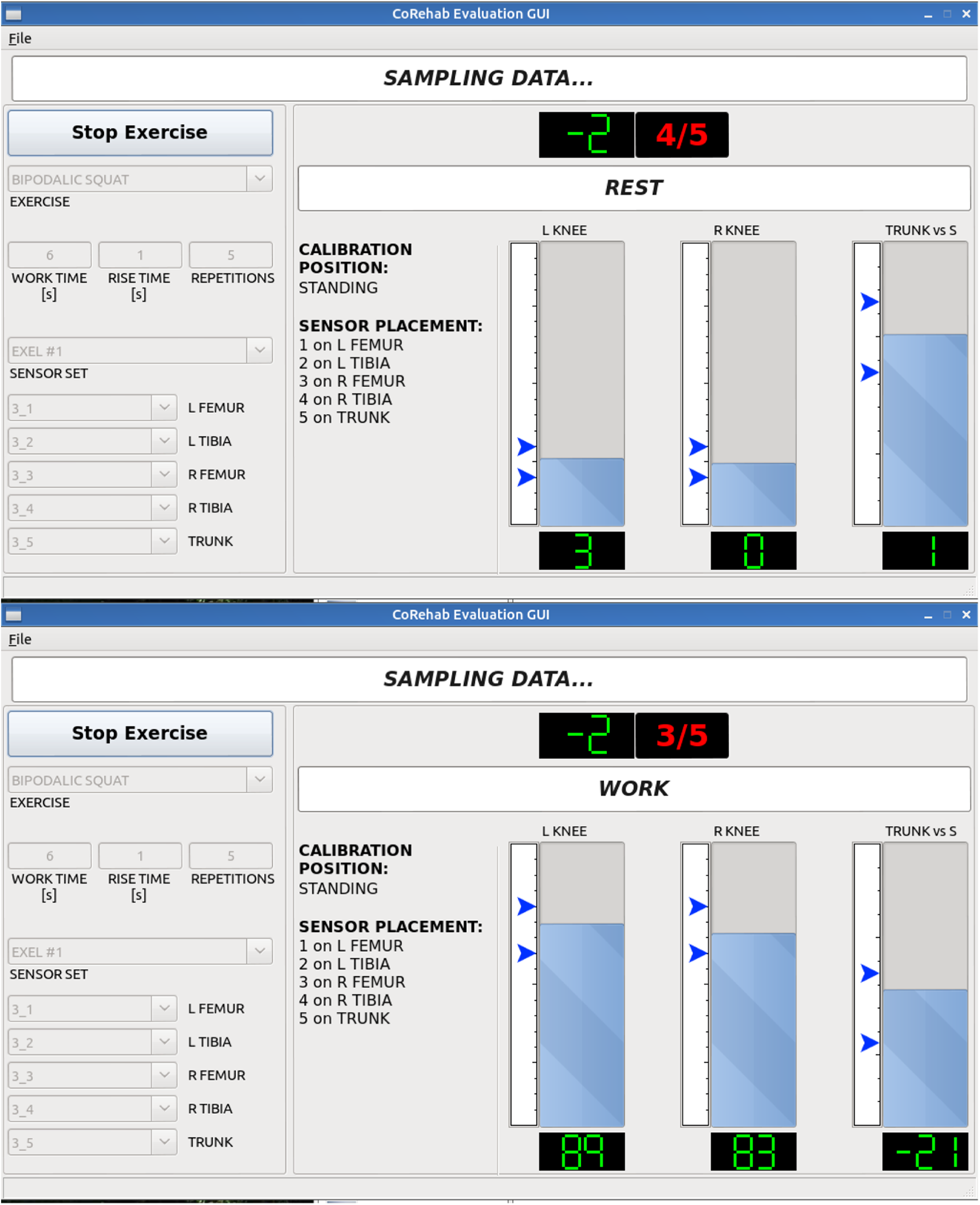

Supplement: Supplementary file 2 — Authors’ original file for figure 2 [file 12984_2014_656_MOESM2_ESM.tif]

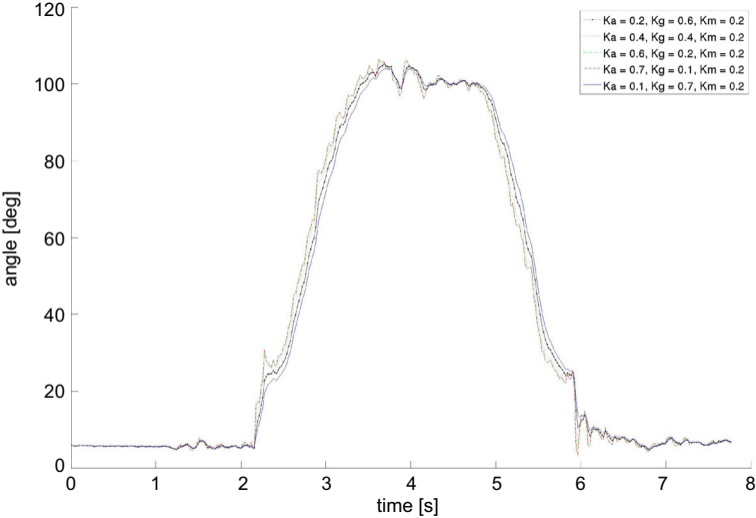

Supplement: Supplementary file 3 — Authors’ original file for figure 3 [file 12984_2014_656_MOESM3_ESM.pdf]

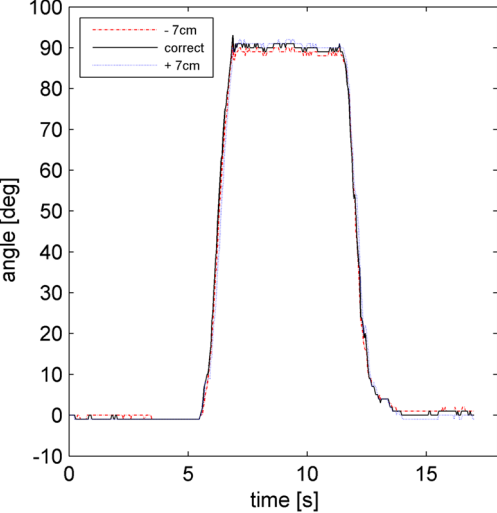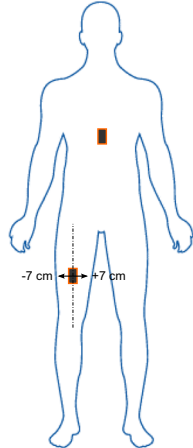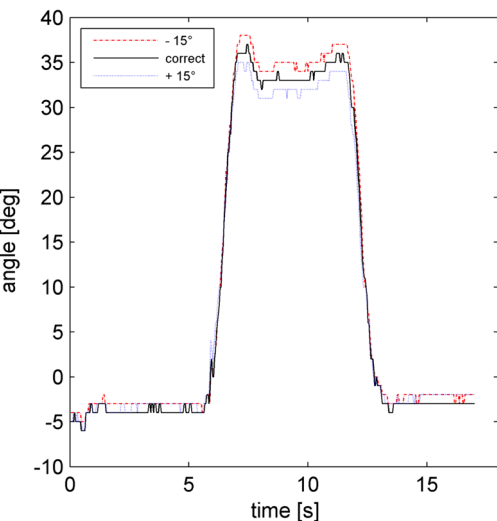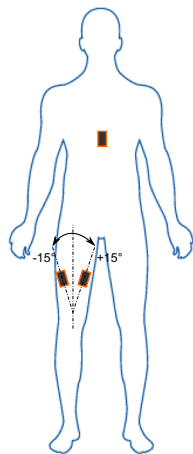

Supplement: Supplementary file 4 — Authors’ original file for figure 4 [file 12984_2014_656_MOESM4_ESM.pdf]

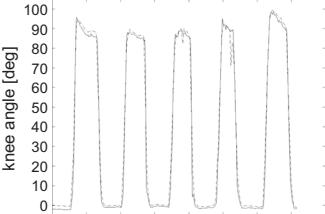

knee flexion

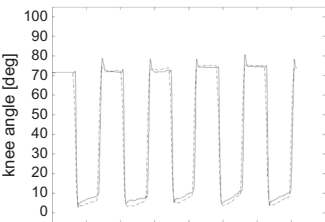

knee extension

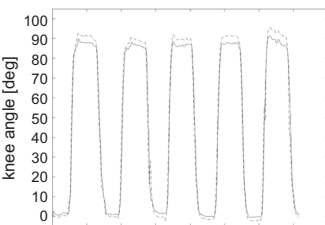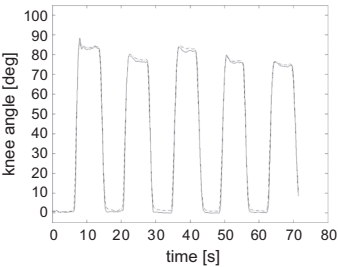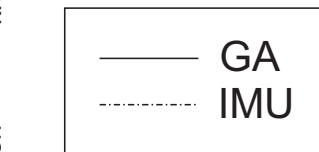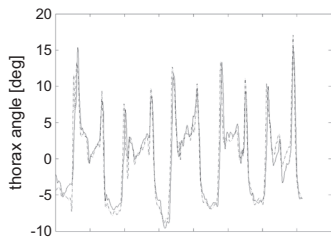

lunge

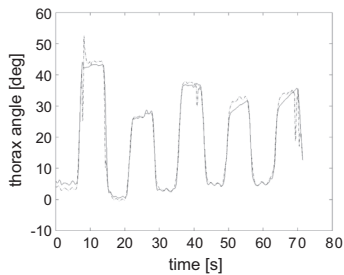

squat

Supplement: Supplementary file 5 — Authors’ original file for figure 5 [file 12984_2014_656_MOESM5_ESM.pdf]
